# The Level of FGF 21 as a New Risk Factor for the Occurrence of Cardiometabolic Disorders amongst the Psoriatic Patients

**DOI:** 10.3390/jcm8122206

**Published:** 2019-12-13

**Authors:** Paulina Kiluk, Anna Baran, Tomasz W. Kaminski, Magdalena Maciaszek, Iwona Flisiak

**Affiliations:** 1 Department of Dermatology and Venereology Medical University of Bialystok, Zurawia 14 St, 15-540 Bialystok, Poland; aannabaran@wp.pl (A.B.); iflisiak@umb.edu.pl (I.F.); 2 Department of Pharmacodynamics, Medical University of Bialystok, Mickiewicza 2C St, 15-222 Bialystok, Poland; tomasz.kaminski@umb.edu.pl; 3 Department of Infectious Diseases and Hepatology Medical University of Bialystok, Zurawia 14 St, 15-540 Bialystok, Poland; mm.maciaszek@wp.pl

**Keywords:** psoriasis, fibroblast growth factor 21, fibroblast growth factor 23, cardiometabolic diseases

## Abstract

Fibroblast growth factors 21 and 23 are used as markers of cardiometabolic disorders which are common comorbidities in psoriasis. The study aimed to evaluate the serum level of these factors in psoriatic patients and elucidate the possible interplay between disease activity, metabolic or inflammatory parameters, and systemic treatment. A total of 33 patients with active plaque-type psoriasis and 11 healthy controls were enrolled in the study. Patients were divided into subgroups based on their BMI, disease severity, and treatment. Blood samples were collected at the beginning of the study and after 3 months of systemic treatment with acitretin or methotrexate. Serum FGF21 levels in psoriatic patients were higher versus control group (*p* < 0.05). FGF21 levels regarding psoriasis activity were significantly increased in all three subgroups compared to the controls (*p* < 0.05). Regarding FGF23, no significant changes were found beside positive correlation with aspartate transferase (*p* < 0.05). No significant effect of systemic treatment on FGF21 and FGF23 levels was found. Interestingly, a nearly threefold decrease in FGF21 concentration after acitretin-based treatment was observed (*p* < 0.05). After methotrexate therapy, FGF21 levels remained unchanged. FGF21 levels might be helpful in prediction of the risk of cardiometabolic comorbidities development especially in patients with severe psoriasis and obesity.

## 1. Introduction

Psoriasis is a common and chronic inflammatory skin disease whose exact pathogenesis remains uncertain and continues to be a topic of extensive research. Nowadays, research focuses not only on understanding the genesis of skin lesions but especially on the explanation of systemic disorders associated with psoriasis. Elevated risk of the occurrence of chronic inflammatory diseases in psoriatic patients, such as inflammatory bowel diseases, nonalcoholic fatty liver disease (NAFLD), diabetes mellitus (DM), obesity, or metabolic syndrome (MS) has been proven in various studies [1]. Additionally, for years, psoriasis has been associated with an increased risk of atherosclerosis and its consequences, such as cardiovascular disease (CAD) or stroke [2,3]. It was even reported that the accelerated development of cardiovascular complications in patients with psoriasis leads to a 5-year reduction in life expectancy [4]. Chronic inflammation is a well-established factor linking both cutaneous manifestation of the disease and the increased coexistence of the abovementioned cardiometabolic disorders. To describe this concept, the term of “psoriatic march” was introduced [5]. 

Understanding of mutual connections between inflammatory state, psoriasis progression and severity, as well as cardiometabolic dysfunction leads to an intensified search for new markers of inflammation that would provide a tool for an early diagnosis of metabolic disorders in psoriatic patients. Moreover, this would play a crucial role in the discovery of new therapeutic methods aimed not only to alleviate skin symptoms but also attenuate systemic complications. Nowadays, the use of fibroblast growth factors, namely 21 and 23 (FGF21, FGF23), are considered as a promising approach to assess the risk and diagnose the presence of psoriasis-related cardiometabolic abnormalities.

The fibroblast growth factor family is composed of 22 members that are involved in a variety of biological functions, including cell growth, development, angiogenesis, and wound healing [6]. FGF21 and FGF23 are members of the ‘endocrine’ FGF subfamily, involved in metabolic regulation which also includes human FGF19 and its murine homolog FGF15 [7,8]. The endocrine FGF subfamily are atypical FGFs due to the lack of affinity for binding heparin and heparin sulfate, which permits them to freely diffuse away from their tissue of origin and serve as endocrine molecules, inducing systemic effects [9]. Paracrine and endocrine FGFs signal through cell surface-localized FGF receptors (FGFRs) belonging to the tyrosine kinase receptor family [6]. Additionally, members of endocrine FGFs subfamily require a co-factor to initiate signaling pathways. FGF15/19 and FGF21 require β-klotho protein as a co-factor, while in the case of FGF23, α-klotho is needed [10,11]. FGF21 is a polypeptide of 210 amino acids expressed predominantly in the liver and, to a lesser extent, in skeletal muscle, adipocytes, and pancreas [12,13,14]. Even though FGFRs are widely distributed in the organism, the tissue-specific effects of FGF21 are limited to those expressing β-klotho, including adipose tissue, liver, pancreas, and specific brain nuclei, like nucleus tractus solitarii, area postrema, suprachiasmatic nucleus, and paraventricular nucleus [15,16,17]. FGF21, distinctly from other FGFs, does not manifest mitogen activity and acts as an endocrine molecule, normalizing glucose and fatty acid (FA) metabolism. Endogenous FGF21 has been demonstrated to act as a stress-responsive hormone to protect against different metabolic or environmental stimuli in pathological conditions [18]. Increased levels of FGF21 were reported under physiological conditions such as starvation, overfeeding, ketogenic diets, low-protein diets, and high-carbohydrate diets [19,20,21,22,23]. The primary functions of circulating FGF21, which is released due to cellular and metabolic stress, are to increase glucose uptake in adipose tissue, augment lipolysis, enhance the production of ketone bodies in the liver, and to regulate energy balance in the body [14,18,24]. Importantly, it was also observed that its circulating levels are often elevated in obesity, dyslipidemia, type 2 diabetes (T2D), and CAD, which suggests its role in the initiation as well as development of metabolic disorders [25]. Thus, FGF21 is reported as a potential therapeutic target and marker for these diseases but the exact mechanism of this link is still unclear [26]. 

Another endocrine FGF that raises more and more interest due to its supposed role in the development of MS and its complications in the circulatory system, is FGF23. It is a 32 kDa protein, predominantly produced by osteocytes and osteoblasts in bone and regulates phosphorus and vitamin D metabolism [27]. It is secreted in response to the levels of active vitamin D, parathyroid hormone (PTH), and elevation in plasma phosphorus concentration. It decreases the conversion of 25(OH)D3 to 1,25(OH)_2_ vitamin D3 by 1-α-hydroxylase enzyme and suppresses the secretion of PTH from the parathyroid gland. FGF23 downregulates sodium and phosphorus co-transport in the proximal tube. It also has been associated with many complications among patients with chronic kidney disease (CKD), particularly, uremic bone disease [28]. Interestingly, FGF23 concentrations have been connected with the occurrence of insulin resistance (IR) as well as inflammatory state, dyslipidemia, and visceral adiposity [29,30].

Despite the existence of a broad spectrum of evidence, the exact role of FGF21 and FGF23 in psoriasis still requires further studies. To the best of our knowledge, this is the first study which evaluates serum FGF21 and FGF23 concentration in patients with active plaque-type psoriasis and the relationship with the disease severity, metabolic or inflammatory parameters, and the influence of systemic treatment. We aimed to investigate whether FGF21 or FGF23 could be useful in predicting the risk of cardiometabolic disorders in psoriatic patients as well as assess the impact of systemic therapy on serum levels of FGF21 and FGF23.

## 2. Materials and Methods

The prospective study was conducted on 33 patients (21 male and 12 female) with exacerbation of plaque-type psoriasis, compared to 11 sex-, age- and BMI-matched healthy controls. We excluded from the study patients with other types of psoriasis, chronic metabolic or inflammatory diseases, malignancy, and pregnancy. Body mass index (BMI) was evaluated as weight/height^2^ (kg/m^2^). Psoriasis area and severity index (PASI) were estimated by the same investigator in all patients. The study group was divided depending on the PASI severity into 3 groups. PASI score under 10 defined psoriasis as mild (PASI 1), between 10 and 20 as moderate (PASI 2), and above 20 as severe (PASI 3). All participants were also divided regarding BMI, where group 0 means the controls, BMI 1 includes 10 normal weight (18.5–24.9) psoriatics, BMI 2 indicates overweight (BMI 25–29.9) and consists of 11 patients, and BMI 3 indicates obesity (BMI > 30), noted in 12 subjects from the study group. Blood samples were collected before starting systemic treatment and repeated after 3 months of therapy. Treatment was carried out with either 15 mg/week methotrexate (MTX) (19 patients) or acitretin (14 persons) at a body weight dose of 0.5 mg/kg/day. All participants gave their signed informed consent before participation. The study was approved by the Bioethical Committee of the Medical University of Bialystok (Protocol number R-I-002/354/2015) and was in accordance with the principle of the Helsinki Declaration.

### 2.1. Serum Collection

Fasting blood samples were collected from the study and control group, after overnight fasting, using Sarstedt S-Monovette tubes and allowed to clot for 30 min before centrifugation for 15 min at 2000× *g*, and then separated serum was frozen immediately and preserved at −80 °C until analysis. Blood samples for biochemical tests and blood counts was collected at the same time to other tubes and were performed by routine laboratory techniques using an automated analyzer. Serum levels of assay parameters were measured using validated and calibrated Bio-Plex 200 System provided by Bio-Rad. In our research, we used Bio-Plex Pro RBM Human Metabolic Panel 2 which includes 8 magnetic bead-based assays to measure among other FGF21 and FGF23.

### 2.2. Statistical Analysis

The normality of obtained distribution was tested using the Shapiro–Wilk test and quantitative data were expressed as mean ± SD. The non-Gaussian data were presented as a median (full range). The Student’s *t* test or nonparametric Mann–Whitney test were used to compare differences between psoriasis group and control group, whereas for binary data, the chi-square test was used. The analysis of variance (ANOVA) was used to check differences between the psoriasis subgroups. The correlations between studied variables were determined by Spearman’s rank correlation analysis. Two-tailed *p* < 0.05 was considered to be statistically significant. Computations were performed using GraphPad 6 Prism Software (GraphPad Software, San Diego, CA, USA). The power of the analysis was estimated using Stata IC 13 Software (Stata Corp LLC, College Station, TX, USA).

## 3. Results

Serum FGF21 and FGF23 levels were analyzed in 33 patients with active plaque-type psoriasis (12 women/21 men) with the mean age of 54 (24–85) and 11 healthy controls matched for age, weight, and BMI. The median BMI of psoriatic patients was 28.86 kg/m^2^ (17.3–43). From the whole group of patients, 10 persons (30.3%) had normal body mass, 11 (33.3%) were overweight, and 12 (36.3%) were obese. Severity of psoriasis expressed by median PASI score was 15 (5.4–32.7) before treatment and 3.4 (0.7–15) after therapy. According to the division of the study group in terms of psoriasis severity, the mild form was present in 8 persons (24.2%), moderate in 13 (39.4%), and severe psoriasis was diagnosed in 12 (36.4%). The demographic, clinical and laboratory characteristic of the study group are summarized in Table 1 and Table 2.

**Figure 1:**
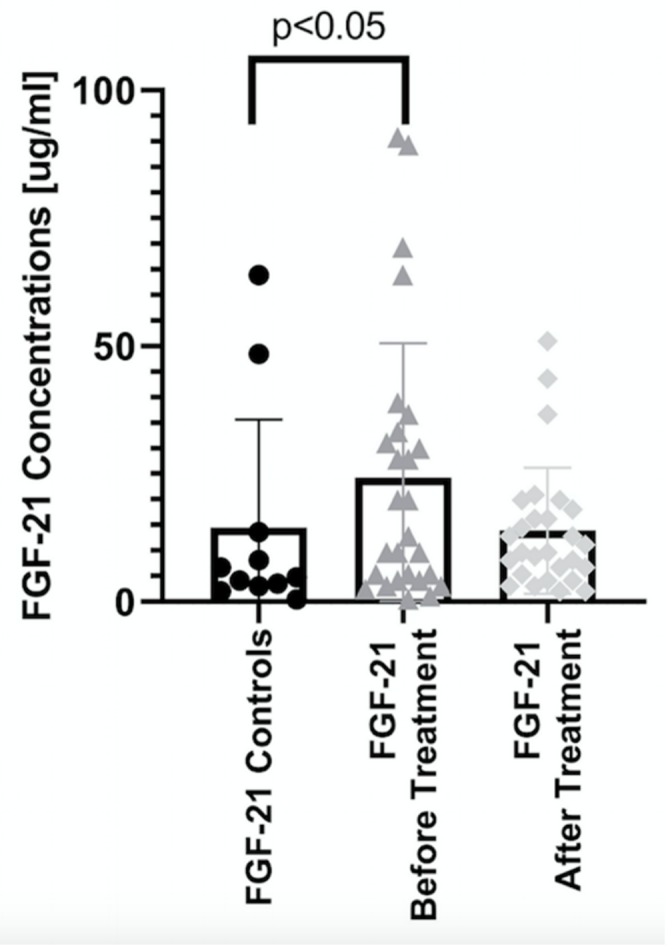
Comparison of the levels of FGF21 between controls and patients before and after treatment.

**Figure 2:**
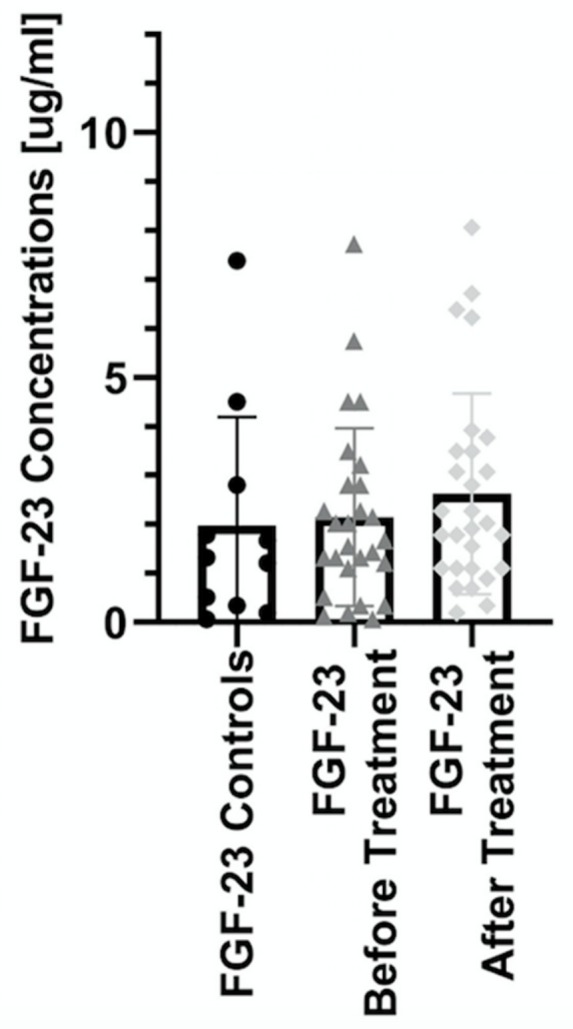
Comparison of the levels of FGF23 between controls and patients before and after treatment.

The median serum FGF21 concentration in psoriatic patients before treatment was 19.9 (0.42–1993) pg/mL and was significantly higher than in control group (4.75 (0.42–63.9)) with a statistical significance of *p* < 0.05 (Figure 1). In the case of FGF23, no significant changes were found between its concentration in patients with psoriasis and controls (Figure 2).

Taking into consideration the BMI subgroups, the highest concentrations of FGF21 were found in obese (BMI 3) psoriatic patients compared to those with normal body weight (BMI 1) or overweight (BMI 2) with values of *p* < 0.01 in both analyses. The concentration was almost three times higher in BMI 3 group. There were no differences between subgroups BMI 1 and BMI 2 (Table 2). Assessing FGF23 depending on BMI, statistically significantly higher concentrations were observed both between individual BMI groups (*p* < 0.01) as well as in relation to the control group (*p* < 0.05). The level of FGF23 was higher in patients with normal body weight, not in obese patients (Table 3).

In the study, we observed positive correlation between FGF21 concentration and severity of psoriasis expressed through PASI on the border of statistical significance (*p* = 0.05). However, when we looked closer, we found significantly (*p* < 0.05) increased FGF21 levels in all three subgroups comparing to the controls. Statistically remarkable differences were also found between the values of FGF21 in these three subgroups. Interestingly, in the group of patients with severe psoriasis, FGF21 level was relevantly high with statistical significance of *p* < 0.05. (Table 4). Serum FGF23 concentrations did not correlate with total PASI score as well as did not respond with psoriasis severity in all three subgroups (Table 4).

After three months of systemic treatment, not only improvement of skin lesions in all studied patients was observed but also limitation of inflammation expressed in decrease of serum concentrations of inflammatory parameters (white blood count, C-reactive protein) (Table 1). FGF21 level did not significantly change after total systemic therapy comparing to the initial concentration (Figure 1). However, there was a statistically significant persistence of the elevated level of FGF21 in treated psoriatic patients in relation to healthy subjects (*p* < 0.05) (Table 3). In case of FGF 23 no relationships were found (Figure 2). Assessing the effect of treatment in BMI subgroups, there was a twofold decrease in FGF21 level after treatment in the obese group, but this decrease did not reach statistical significance. Interestingly, a statistical significant reduction in FGF21 level was observed after total treatment in patients with severe psoriasis as shown in Table 4. There were no important differences in FGF23 concentrations in three PASI or BMI subgroups after treatment (Table 3 and Table 4). After division into subgroups of patients treated with both drugs separately, FGF21 level decreased after acitretin more than threefold with statistical significance of *p* < 0.05 as shown in Table 5. After methotrexate treatment its level remained the same as before. There were no relevant changes in FGF23 levels after systemic treatment, neither acitretin nor methotrexate (Table 5).

We have also correlated serum of FGF21 and FGF23 with biochemical, inflammatory, and blood count results. In relation to the whole group of patients with psoriasis, no significant correlations were found between serum FGF21 and 23 and laboratory parameters, except for the statistically significant relationship between the pre-treatment FGF21 level and the CRP value (*p* = 0.01). When we looked closer at the relationships in individual subgroups, we also found some correlations. In the group of patients with normal body weight, we found no correlation of FGF21 or FGF23 with laboratory results. However, in the group of overweight patients, we noticed statistically significant positive correlations of pre-treatment FGF21 concentration with CRP (*p* < 0.05), platelets (PLT) (*p* < 0.05), and negative correlations with hemoglobin (HGB) (*p* < 0.05) and red blood cells (RBCs) (*p* < 0.05) levels. In this group of patients, the level of pre-treatment FGF23 correlated statistically significantly with the concentration of triglycerides (TG) (*p* < 0.05) and PLT (*p* < 0.05). Among obese patients, we showed statistically significant correlation of FGF 21 with total cholesterol (*p* < 0.05). There was no correlation in all BMI subgroups in relation to the FGFs values after treatment. Similarly to the group of patients with normal body mass, also in the group with the least-expressed skin lesions (PASI < 10), no significant dependence between FGF21 or FGF23 and the results of laboratory tests was observed. In patients with moderate psoriasis, a significant (*p* < 0.05) negative correlation was demonstrated between the level of pre-treatment FGF21 and FGF23, and RBCs and HGB. Among patients with severe psoriasis, a similar trend of FGF21 before treatment was shown with HGB and RBCs concentrations, respectively *p* = 0.06 and *p* < 0.05. Interestingly, after treatment in this group of patients, the level of FGF21 was significantly negatively correlated with the concentration of leukocytes (white blood count, WBC) (*p* < 0.05) and glucose (*p* < 0.05). FGF23 values both before and after treatment significantly correlated with AST (aspartate transaminase) (*p* < 0.05). Among patients treated with acitretin, there was a positive correlation between pre-treatment FGF21 and CRP (*p* < 0.05) and negative with total cholesterol (*p* < 0.05). FGF23 correlated negatively with total cholesterol (*p* < 0.01), triglycerides (*p* < 0.01) and positively with AST (*p* < 0.05). Loss of these dependencies was noted after therapy with acitretin. In the group of patients treated with MTX, there were no significant relationships between FGFs and laboratory results.

## 4. Discussion

In recent years, much more attention has been paid to more frequent coexistence of metabolic disorders such as type 2 diabetes, dyslipidemia, obesity, as well as cardiovascular diseases in psoriatics. Thus, psoriasis is more often considered as an independent risk factor accelerating the development of CMDs, leading to increased mortality and morbidity amongst these patients compared to general population [31]. The presence of comorbidities in the patient is also important when selecting the appropriate treatment method. Currently, we can choose from both conventional and biological agents.

On the other hand, pre-existing obesity or MS increase the risk of development of psoriasis due to the chronic, systemic metabolic inflammatory state, termed metaflammation [31]. Thus, a closer look at factors involved in this interplay can help in the early diagnosis and treatment of cardiometabolic disorders. One such factor could be FGF21, which is one of the crucial regulators of homeostasis of lipids, glucose, and energy metabolism. The involvement of FGF21 in these metabolic pathways also reflects its contribution to the development of IR, DM, or MS. Moreover, the increase in FGF21 level is induced by pathological physical stress conditions like obesity, anorexia nervosa, hypothermia, amino acid deprivation or malnutrition, and nephropathy [32,33,34,35,36,37,38].

To date, there are no papers describing the concentrations of FGF21 in psoriasis. In this study, we demonstrate, for the first time, elevated serum FGF21 concentrations in psoriatic patients compared to healthy controls. This leads to the question if psoriasis can also be considered as a state of metaflammation and if FGF21 could be a novel biomarker of psoriasis or progression of its comorbidities. Due to the lack of other studies among patients with psoriasis, we can only refer to publications highlighting the presumed involvement of FGF21 in the development of CMDs, and thus emphasize the possible association of psoriasis with the accelerated development of these complications. As an example, Lin Z et al. revealed that serum levels of FGF21 are increased in subjects with coronary heart disease (CHD) and could be used as a biomarker [39]. They also suggested that serum concentrations of FGF21 in humans are not related to insulin secretion but, rather, to lipid metabolism. In our study, we noticed a positive correlation between pre-treatment FGF21 and cholesterol levels in obese psoriatics. This observation does not only reflect the possible role of FGF21 in lipid metabolism, but also suggests an increased risk of developing lipid disorders, including atherosclerosis and CVD in these patients. The disappearance of this correlation after treatment underlines the possible reduction of the risk in obese psoriatics. Thus, this confirms that the implementation of systemic treatment in obese patients is reasonable, regardless of the severity of psoriasis. Furthermore, Shen et al. recognized serum FGF21 level as an independent risk factor of coronary artery disease [40]. Recent studies have also prompted a possible role of FGF21 in atherosclerosis which in turn is highly related to psoriasis. Chow W.S. et al. noted that elevated FGF21 were associated with carotid intima–media thickness in humans, independent of CVD risk factors [41]. 

It is not only cardiac complications that are associated with elevated FGF21. There are several studies that confirmed significantly higher levels of circulating FGF21 in patients with T2D than in healthy controls. Moreover, the level of FGF21 correlated with insulin resistance [42,43,44]. Altered, higher concentration of FGF21 has also been reported in patients with NAFLD versus control subjects [21,45]. Interestingly, Li H. et al. also noted that FGF21 measurement may be helpful in detection of mild steatosis [45]. NAFLD is a manifestation of abnormal metabolism within the liver resulting in the accumulation of triglycerides within the hepatocytes. Risk factors include male gender, age, obesity, IR, and MS [46]. There are many studies confirming the higher incidence of NAFLD in patients with psoriasis. Additionally, it was found that psoriatic patients had more severe liver disease than the non-psoriatic ones. On the other hand, psoriatic patients with NAFLD had more severe and longer duration of skin disease than those without NAFLD [47,48,49]. 

FGF21 has also been found to be related to renal dysfunction. Lin Z. et al. have shown that FGF21 levels become increased with the development of early to end stage of chronic kidney disease, and are independently associated with the loss of renal function [38]. These findings support the possible participation of FGF21 in the development of metabolic or perhaps renal disorders in patients with psoriasis, and could be a possible marker for their progression.

When we looked more closely at the studied subgroups of our patients, we found with statistical significance three times higher FGF21 concentration in obese psoriatics. In the literature, there are similar reports with elevated levels of FGF21 in obese subjects [32]. Moreover, in a longitudinal one-year follow-up study of obese children who started a weight loss program, Reinehr T. et al. noted not only significant correlation between FGF21 levels and BMI but also a decrease in its concentration during weight loss [50]. Thus, it emphasizes the link between the molecule and metabolic inflammation also in psoriasis but also encourages deeper research on diet restriction impact on FGF levels in psoriatics.

The association of higher FGF21 serum levels with all mentioned components of MS may be a compensatory protective response due to the underlying metabolic stress in all these disorders. On the other hand, it may be due to FGF21 resistance, in which impaired interactions of FGF21 with its receptor and downregulation of downstream signaling pathways results in the need for supraphysiological doses of FGF21 to achieve its protective physiological function [25]. These findings are supported also by Li H. et al. who proposed that elevated levels of endogenous FGF21 in obesity serve as a defense mechanism to protect against systemic insulin resistance [51]. However, this issue remains debatable and requires further research. Significantly higher levels of FGF21 in obese patients with psoriasis can result from both the accumulation of adipose tissue as well as that this increase can be enhanced by the immune-mediated inflammation in psoriasis. Similarly, this inflammation and its severity may be responsible for elevated FGF21 concentrations among patients with a severe form of psoriasis, which we have presented in our research. Due to lack of data from the literature, it is impossible to compare our outcomes. Hence, we can only point out the fact that increased FGF21 observed in all three groups of psoriatics, from mild to severe, suggests that systemic inflammation due to psoriasis favored FGF21 production. Thus, FGF21 can be not only a marker of coexisting metabolic disorders in psoriasis but also a marker of the progression and severity of skin lesions.

Moreover, the possible contribution of FGF21 to the development and maintenance of inflammation are underlined by the correlation observed in our research between the concentration of FGF21 in patients with psoriasis and the CRP value. CRP as well as FGF21 are produced in the liver and in adipocytes, which is connected with the acute-phase response pathway and plays a key role in destabilization and rupture, leading to acute cardiovascular events [52]. Multiple findings have established that increased CRP levels are associated with increased CHD [52,53]. The correlation between these parameters, demonstrated in our study, indicates the existence of possible pathogenetic connections, thus suggesting the involvement of the molecule in the development of CVD and metaflammation. Hence, elevated FGF21 levels in patients with psoriasis may be a good predictive marker of CMDs in psoriatics. Moreover, our study showed a negative correlation of pre-treatment FGF21 levels with RBC count and HGB, mainly in patients with moderate to severe psoriasis. The results obtained are consistent with the findings of Rocha-Pereira P. et al. who showed a reduced number of RBCs in psoriatic patients, which was connected with worsening of psoriasis [54]. 

The innovation of this study was also the assessment of the impact of systemic treatment used in psoriasis on the level of FGF21. Currently, the first-line drugs for the treatment of severe psoriasis are still very often acitretin and methotrexate. Hence, their use in our study. Despite having known about their beneficial effects on skin lesions for many years, their impact on metabolic disorders in psoriasis is not yet fully understood. After three months of systemic treatment with acitretin or MTX, we did not observe a significant decrease in FGF21 concentration. Moreover, its level remained statistically higher in patients with psoriasis after treatment compared to healthy control. This might suggests the lack of significant treatment effects on FGF21 production or, presumably, resistance of the molecule as a compensation, or other underlying confounding mechanisms. Interestingly, when we divided patients into groups depending on the drug used, we observed almost threefold decrease in FGF21 concentration in the acitretin group, while the concentration of the factor in the MTX group remained almost the same. This observation is all the more interesting because it is believed that the use of methotrexate in psoriasis reduces the risk of CVD and MI by limiting chronic inflammation. A possible explanation for our observations is the lack of influence of methotrexate on signaling pathways and secretion of FGF21. In turn, the use of acitretin, which is often associated with the development of sides effects as hyperlipidemia, may contribute to the development of cardiometabolic complications in these patients. The observed threefold decrease in FGF21 during treatment with acitretin, although not statistically significant but remarkable, suggests the existence of a link between FGF21 and the systemic action exerted by acitretin. This interplay is also reflected by observations in our study through correlations with inflammatory or metabolic indices which disappeared after acitretin therapy. The results suggest a possible protective effect of acitretin and perhaps FGFs on patients with psoriasis on the development of CMDs. These observations, as well as those concerning methotrexate, will certainly require further research on a larger group of patients. When analyzing the effect of treatment on the level of FGF21 in the serum depending on the body weight of the patients, only a two-fold decrease was observed in the group of obese ones. However, this decrease was not statistically significant. A significant decrease in FGF21 concentration after systemic treatment was observed in patients with severe psoriasis. These results may support presumptive use of FGF21 as a marker of systemic therapy effectiveness in the group of patients with the most severe form of psoriasis.

With regard to FGF23 serum level, we have not seen any correlation between healthy controls and psoriatic patients, as well as after systemic treatment in total and separately. Our observations are inconsistent with reports presented by Okan et al., who noted significantly higher concentration of FGF23 in psoriatic patients than healthy controls [55]. Moreover, they suggest putative interactions between FGF23 and inflammatory regulatory pathways due to its association with comorbidities of psoriasis like insulin resistance, dyslipidemia, atherosclerosis, or markers of chronic inflammation like interleukin-6, CRP, TNFα, and fibrinogen. However, the observed positive relation with AST activity might point to a potential role of FGF23 in liver activity in psoriatics. The relationship between FGF23 concentration and liver function has already been pointed out by Prié D. et al. According to them, chronic liver lesions can induce expression of FGF23 mRNA, leading to increased FGF23 concentration, which is related to a higher mortality in patients on a liver transplant waiting list [56]. Our initial research has not confirmed the possibility of using FGF23 as a marker for CVD development in psoriatic patients. 

The limitations of our study may result from a small number of patients included and an incomplete control group, although the groups were relatively uniform in terms of biochemical and clinical characteristics. Moreover, additional statistical analysis revealed the existence of many divergent correlations between analyzed FGF molecules and morphological parameters in both, all-patient group, as well as when divided into subgroups, as shown in Tables S1 and S2. Due to the extensive amount of statistical data, these data were not included in the publication and are available from the corresponding author on request.

## 5. Conclusions

This is the first prospective study assessing serum concentrations of FGF21 and FGF23 in psoriatic patients and adds value by evaluating the relationships with systemic medications such as acitretin or methotrexate. 

Numerous studies have confirmed the contribution of FGFs to the development of CMDs which are closely associated with psoriasis. Endogenous FGF21 acts as a stress-responsive hormone to defend against different metabolic or environmental stress in diverse conditions. Our findings showed significantly increased levels of FGF21 in psoriatic versus healthy controls. We also noticed a tendency to increase of FGF21 level within the psoriasis activity. Furthermore, the highest concentrations were noted in obese patients and subjects with the most severe form of psoriasis. Moreover, we showed a positive correlation of FGF21 concentration with CRP, pointing to its potential role as an indicator of metabolically derived inflammation in psoriatics. Notable associations with morphotic blood elements highlights the unknown relationship of FGF21 with anemia or other hematological disturbances in psoriasis. All these findings emphasize the possibility of using FGF21 as a novel biomarker for severity of skin lesions, as well as imply the careful observation of these group of patients for accelerated development of cardiometabolic disorders in which FGF21 may participate. The observed elevated concentration of FGF21 in patients with psoriasis also raises the question of whether psoriasis can be treated as a state of metabolic stress in the body, strong enough to trigger an increased production of FGF21. Additionally, we unexpectedly noticed that treatment with acitretin decreases the level of FGF21 and cardiometabolic parameters more than methotrexate. Interestingly, for the first time, we point out the existence of associations between FGF21 and FGF23 with blood count parameters as well as markers of inflammation and metabolic condition indicators. However, these observations require further extended research on a larger group of patients. In our study, we have not shown any changes in serum levels of FGF23 in psoriatic patients. However, positive correlation with aspartate aminotransferase activity might point to a possible link with liver activity. In addition, the observed increase in FGF21, depending on the severity of psoriatic lesions, makes FGF21 an interesting marker combining the progression of psoriasis with an increased risk of developing metabolic disorders.

## Figures and Tables

**Table 1:** Baseline characteristic of control group and patients.

Parameter	Controls (*n* = 11)	Patients (*n* = 33)
Age (years)	54.4 ± 9.11	54.2 ± 16.8
Weight (kg)	69.4 ± 15.2	84.8 ± 20.9 *
Height (cm)	166 ± 8.83	171 ± 10
BMI	25 ± 3.59	28.9 ± 6.42 *

* statistical significance with *p* < 0.05, between Controls and Patients groups.

**Table 2:** Comparison of inflammation, morphological, and metabolic parameters at the start of the study as well as after treatment.

Parameter	Before Treatment	After Treatment	After Acitretin	After Methotrexate
PASI	15 (5.4–32.7)	3.4 (0.7–15) ^^^	3.4 (0.7–15)	2.6 (1–7.6)
Hemoglobin (mg/dL)	13.6 ± 1.66	13.2 ± 1.45	13 ± 1.5	13 ± 1.16
Red Blood Cells (× 10^3^/mL)	4.38 ± 0.56	4.28 ± 0.47	4.3 ± 0.46	4.2 ± 0.32
White Blood Cells (× 10^3^/mL)	7.04 (4.92–12)	6.42 (4.2–10.3) ^	6.44 (4.2–8.9)	6.4 (4.3–10.34)
Platelets (× 10^3^/mL)	251 ± 73.2	231 ± 59.5	238 ± 62.2	238 ± 62.4
Glucose (mg/dL)	84 (53–215)	87 (55–140)	86 (55–110)	90 (65–140)
C-Reactive Protein (mg/L)	5.13 (1–34.7)	1.87 (0.5–15) ^^	2.37 (1–13.2)	1.6 (0.5–5.8)
Alanine Aminotransferase (IU/L)	24 (9–48)	19 (9–49)	19 (9–42)	23 (12–49)
Aspartate Aminotransferase (IU/L)	21 (14–86)	19 (12–52)	20 (12–39)	20 (13–52)
Total Cholesterol (mg/dL)	170 ± 39.1	168 ± 38.6	170 ± 39.6	165 ± 31.7
Triglycerides (mg/dL)	137 ± 63.1	120 ± 54.6	124 ± 58	125.3 ± 53.9

^/^^/^^^ statistical significance with *p* < 0.05/0.01/0.0001, respectively, between group Before Treatment and After Treatment.

**Table 3:** Comparison of the levels of FGF21 and FGF23 between controls and patients before and after treatment, including differentiation regarding BMI-class. FGF21and FGF23 concentrations given in pg/mL.

	Controls	Patients	BMI I	BMI II	BMI III
**FGF 21**					
Before	4.75 (0.42–63.9)	19.9 (0.42–1993) *	9.6 (1.12–699) * #	9.6 (2.49–197) * #	28.9 (0.42–1993) * #
After		12 (2–11507) *	12.8 (4.15–11507) *	9.6 (2–50.9) *	13.6 (3.02–205) *
**FGF 23**					
Before	1.32 (0.06–7.38)	1.85 (0.06–10.4)	3.21 (1.43–10.4) * #	1.21 (0.12–2.8) * #	2.09 (0.06–7.72) * #
After		1.97 (0.19–8.06)	2.29 (0.19–6.71)	1.44 (0.34–6.38)	2.28 (0.89–8.06)

* statistical significances with *p* < 0.05 between Controls and Patients groups. # statistical significances with *p* < 0.05 between BMI-subgroups.

**Table 4:** Comparison of the levels of FGF21 and FGF23 between controls and patients before and after treatment, including PASI-scoring. FGF21 and FGF23 concentrations given in pg/mL.

	Controls	Study Group	PASI I	PASI II	PASI III
**FGF 21**					
Before	4.75 (0.42–63.9)	19.9 (0.42–1993) *	14.7 (3.02–90.8) * #	7.49 (0.42–63.9) * #	34.9 (3.02–1993) * #
After		12 (2–11507) *	14.5 (2–11507) *	8.49 (2–43.6) *	13.6 (3–2493) *
**FGF 23**					
After	1.32 (0.06–7.38)	1.85 (0.06–10.4)	2.03 (0.12–7.72)	1.43 (0.19–10.4)	1.85 (0.06–5.74)
Before		1.97 (0.19–8.06)	3.49 (0.19–8.06)	1.78 (0.34–6.38)	1.85 (0.89–6.71)

* statistical significances with *p* < 0.05 between Controls and Patients groups. # statistical significances with *p* < 0.05 between BMI-subgroups.

**Table 5:** Comparison of the levels of FGF21 and FGF23 between controls and patients before and after treatment, including differentiation regarding used type of the treatment. FGF21 concentrations given in pg/mL.

	Controls	Study Group	Acitretin	Methotrexat
**FGF 21**				
Before Treatment	4.75 (0.42–63.9)	19.9 (0.42–1993) *	37.7 (1.12–1993) *	11.2 (0.42–89.3) #
After Treatment	-	12 (2–11507)	12 (3.02–2493) *	11.2 (2–11507)
**FGF 23**				
Before Treatment	1.32 (0.06–7.38)	1.85 (0.06–10.4)	2.03 (0.34–10.4)	1.67 (0.06–7.72)
After Treatment	-	1.97 (0.19–8.06)	1.91 (0.69–6.71)	2.28 (0.19–8.06)

* statistical significances with *p* < 0.05 between Controls and Patients groups. # statistical significances with *p* < 0.05 between ACE and MTX before treatment.

## Supplementary Materials

The following are available online at https://www.mdpi.com/2077-0383/8/12/2206/s1, Table S1: Comparison of inflammation, morphological, and metabolic parameters at the start of the study as well as after treatment regarding BMI-scoring., Table S2: Comparison of inflammation, morphological, and metabolic parameters at the start of the study as well as after treatment regarding PASI-scoring.
