# Supplementary material for: The Level of FGF 21 as a New Risk Factor for the Occurrence of Cardiometabolic Disorders amongst the Psoriatic Patients
Source: J Clin Med. 2019 Dec 13;8(12):2206. doi: 10.3390/jcm8122206 (PMC6947480; doi:10.3390/jcm8122206)
Supplement: Supplementary file 1 [file jcm-08-02206-s001.pdf]

Table S1. Comparison of inflammation, morphological, and metabolic parameters at the start of the study as well as after treatment regarding BMI-scoring.

	**Before Treatment**			**After Treatment**		
**Parameter**	**BMI I (*n* = 10)**	**BMI II (*n* = 11)**	**BMI III (*n* = 12)**	**BMI I (*n* = 10)**	**BMI II (*n* = 11)**	**BMI III (*n* = 12)**
PASI	15.1 ± 6.53	17.3 ± 7.83	18.7 ± 8.91	2.5 (1.8–15)^^^	5.4 (1.8–12.5)^^	3.85 (0.7–8.4)^^
Hemoglobin [mg/dl]	13.7 ± 1.53	13.2 ± 1.87	13.8 ± 1.62	13.1 ± 1.7	13.2 ± 1.73	13.2 ± 1.01
Red Blood Cells [x10³/mL]	4.3 ± 0.49	4.29 ± 0.64	4.52 ± 0.56	4.17 ± 0.43	4.34 ± 0.6	4.33 ± 0.39
White Blood Cells [x10³/mL]	7.18 ± 2.12	6.87 ± 1.4	8.33 ± 1.87	6.1 ± 1.42	6.6 ± 1.32	7.08 ± 1.94
Platelets [x10³/mL]	249 ± 83.8	257 ± 76.5	247 ± 66.8	222 ± 75.9	246 ± 47.8	225 ± 56.5
Glucose [mg/dl]	80 (53–215)	80 (69–99)	**99.5 (53–323) ***	86 (55–112)	87 (78–97)	97.5 (65–140)
C-Reactive Protein [mg/L]	3.67 (1–16.1)	4.07 (1–28.6)	7.95 (1.48–34.7)	**1.2 (0.5–2.4) ^**	3.2 (1–15)	**2.4 (1–6) ^^**
Alanine-Aminotransferase [IU/L]	19.9 ± 9.87	17.9 ± 6.82	**33 ± 12.1 ****	17.1 ± 4.07	16.5 ± 6.06	**28.2 ± 10.7 ****
Aspartate-Aminotransferase [IU/L]	20 (15–86)	19 (14–27)	23.5 (15–47)	18.5 (14–39)	16 (12–28)	21 (13–52)
Total Cholesterol [mg/dl]	158 ± 47.5	168 ± 31.6	183 ± 37.1	156 ± 43.5	180 ± 41.2	167 ± 31.3
Triglicerydes [mg/dl]	110 ± 76.8	133 ± 48.4	**165 ± 55.8 ***	101 ± 64.7	127 ± 58.8	**130 ± 40.2 ^**

*/**—statistical significances with *p* values <0.05/0.01, respectively between subgroups of BMIs. ^/^^/^^^—statistical significances with *p* values <0.05/0.01/0.0001S, respectively between group Before Treatment and After Treatment.

Table S2. Comparison of inflammation, morphological, and metabolic parameters at the start of the study as well as after treatment regarding PASI-scoring.

	**Before Treatment**			After Treatment		
**Parameter**	**PASI I**	**PASI II**	**PASI III**	PASI I	PASI II	PASI III
PASI	8.75 ± 1.92	14.1 ± 2.68***	26.1 ± 4.05***###	2.95 ± 1.9 ^^^	3.92 ± 3*^^^	6.3 ± 3.51*#^^^
Hemoglobin [mg/dl]	14.4 ± 1.34	13.7 ± 1.49	12.8 ± 1.83	13.5 ± 0.92	13.3 ± 1.93	12.7 ± 1.06
Red Blood Cells [x10³/mL]	4.61 ± 0.48	4.35 ± 0.61	4.25 ± 0.55	4.33 ± 0.32	4.27 ± 0.62	4.27 ± 0.39
White Blood Cells [x10³/mL]	7.62 ± 2.24	7.35 ± 1.96	7.57 ± 1.67	6.64 ± 1.65	6.4 ± 1.32	6.86 ± 1.92
Platelets [x10³/mL]	230 ± 41.6	257 ± 68.7	258 ± 94.5	226 ± 59.7	239 ± 63.4	226 ± 59.5
Glucose [mg/dl]	94.5 (78–135)	83 (69–215)	82 (53–123)	88.5 (81–125)	86 (65–120)	89 (55–140)
C-Reactive Protein [mg/L]	2.08 (1–7.3)	4 (1–7.2)	15.9(5.13–34.7)***##	1.45 (1–2.46)	1.3 (0.5–13.2)^	4.11 (1–15)*#^^^
Alanine-Aminotransferase [IU/L]	26 ± 13.3	18.1 ± 6.2	29.1 ± 13.5	20.3 ± 6.92	19 ± 5	23.5 ± 13.5
Aspartate-Aminotransferase [IU/L]	21.1 ± 4.36	20.6 ± 4.89	30.3 ± 19.8	20.1 ± 5.91	19.5 ± 4.67	24.4 ± 12.8
Total Cholesterol [mg/dl]	177 ± 43.4	173 ± 46.3	163 ± 28.5	159 ± 35.6	168 ± 51.8	173 ± 22.4
Triglicerydes [mg/dl]	168 ± 73.5	134 ± 69.7	121 ± 43.6	135 ± 54.3	121 ± 69	109 ± 36.1

*/**/***—statistical significances with *p* values <0.05/0.01/0.0001, respectively between PASI I and other subgroups of PASI. #—statistical significances with *p* values <0.05 between PASI II and PASI III. ^/^^/^^^—statistical significances with *p* values <0.05/0.01/0.0001S, respectively between group Before Treatment and After Treatment.
